# Synchronized Intracranial Electrical Activity and Gait Recording in Parkinson’s Disease Patients With Freezing of Gait

**DOI:** 10.3389/fnins.2022.795417

**Published:** 2022-03-03

**Authors:** De-Feng Liu, Bao-Tian Zhao, Guan-Yu Zhu, Yu-Ye Liu, Yu-Tong Bai, Huan-Guang Liu, Yin Jiang, Xin Zhang, Lin-Shi, Hua Zhang, An-Chao Yang, Jian-Guo Zhang

**Affiliations:** ^1^Department of Neurosurgery, Beijing Tiantan Hospital, Capital Medical University, Beijing, China; ^2^Department of Functional Neurosurgery, Beijing Neurosurgical Institute, Capital Medical University, Beijing, China; ^3^Beijing Key Laboratory of Neurostimulation, Beijing, China

**Keywords:** synchronization, intracranial electrical activity, Parkinson’s disease, freezing of gait, motion capture

## Abstract

**Background:**

This study aimed to describe a synchronized intracranial electroencephalogram (EEG) recording and motion capture system, which was designed to explore the neural dynamics during walking of Parkinson’s disease (PD) patients with freezing of gait (FOG). Preliminary analysis was performed to test the reliability of this system.

**Methods:**

A total of 8 patients were enrolled in the study. All patients underwent bilateral STN-DBS surgery and were implanted with a right subdural electrode covering premotor and motor area. Synchronized electrophysiological and gait data were collected using the Nihon Kohden EEG amplifier and Codamotion system when subjects performed the Timed Up and Go (TUG) test. To verify the reliability of the acquisition system and data quality, we calculated and compared the FOG index between freezing and non-freezing periods during walking. For electrophysiological data, we first manually reviewed the scaled (five levels) quality during waking. Spectra comprising broadband electrocorticography (ECoG) and local field potential (LFP) were also compared between the FOG and non-FOG states. Lastly, connectivity analysis using coherence between cortical and STN electrodes were conducted. In addition, we also use machine learning approaches to classified FOG and non-FOG.

**Results:**

A total of 8 patients completed 41 walking tests, 30 of which had frozen episodes, and 21 of the 30 raw data were level 1 or 2 in quality (70%). The mean ± SD walking time for the TUG test was 85.94 ± 47.68 s (range: 38 to 190.14 s); the mean ± SD freezing duration was 12.25 ± 7.35 s (range: 1.71 to 27.50 s). The FOG index significantly increased during the manually labeled FOG period (*P* < 0.05). The beta power of STN LFP in the FOG period was significantly higher than that in the non-FOG period (*P* < 0.05), while the band power of ECoG did not exhibit a significant difference between walking states. The coherence between the ECoG and STN LFP was significantly greater in high beta and gamma bands during the FOG period compared with the shuffled surrogates (*P* < 0.05). Lastly, STN-LFP band power features showed above-chance performance (*p* < 0.01, permutation test) in identifying FOG epochs.

**Conclusion:**

In this study, we established and verified the synchronized ECoG/LFP and gait recording system in PD patients with FOG. Further neural substrates underlying FOG could be explored using the current system.

## Introduction

Parkinson’s disease (PD) is a degenerative disease of the nervous system that occurs in older people. Freezing of gait (FOG) is a type of gait disorder characterized by recurrent short-term gait delays and cessation, which can appear suddenly during stepping or walking (Walton et al., 2015). As PD progresses, the incidence of FOG gradually increases, and the incidence over 10 years is as high as 58% (Giladi et al., 2001). FOG is a disability that is the main cause of falls in patients with PD, significantly hinders the activity and autonomy of patients’ daily life, and greatly affects the quality of life of patients. At present, there is a lack of specific drugs for FOG symptoms, and the efficacy of levodopa and amantadine in the treatment of FOG remains controversial (Macht et al., 2007; Giladi, 2008).

In recent years, with the development of multi-modal imaging technology and electrophysiological technology, more attention has been paid to the neural mechanisms of FOG. It is believed that the abnormal function of the basal ganglia–cortical loop may play an important role in the occurrence and development of FOG (Bartels and Leenders, 2008; Snijders et al., 2016). For example, Lv et al. (2021) examined the association between basal ganglia perivascular spaces and FOG using high resolution 7T-magnetic resonance imaging (MRI), and found that basal ganglia perivascular spaces were significantly greater during frozen episodes. Although neuroimaging studies provide adequate coverage and spatial resolution, they do not reflect the dynamic response to FOG events.

Electroencephalogram (EEG) studies are advantageous in depicting the neural dynamics underlying FOG. In previous studies on electrophysiology of FOG, many scholars recorded scalp EEG of PD-FOG patients during walking, and some scholars also used gait analysis (Handojoseno et al., 2015; Günther et al., 2019; Brugger et al., 2020; Asher et al., 2021; Cao et al., 2021; Stuart et al., 2021). The results reported the characteristics of EEG dynamics and the coupling of different cortical locations of PD-FOG. However, these studies have some limitations. First, they did not obtain information on subcortical structures, and second, they did not use synchronized gait analysis to accurately distinguish whether FOG durations. Other scholars have recoded the LFP of the subcortical STN in DBS patients during walking, and combined gait analysis simultaneously, reporting the relationship between the features of STN and the occurrence of FOG (Fischer et al., 2018; Chen et al., 2019). At present, there are few researches on the combination of multi-target electrophysiological signal recording and gait analysis. Pozzi et al. (2019) added scalp EEG on above basis and reported the derangement of locomotor network dynamics in PD-FOG. However, scalp EEG cannot eliminate the attenuation effect of the scalp, skull, dura, and other structures on electrical activity, limiting its efficacy on spatial resolution and discernibility of some frequency components. In our study, the electrodes were placed under the dura to ameliorate this shortcoming. Our method platform still achieved synchronous record of ECoG, STN-LFP and gait data, which means that gait data characteristics can be used to accurately distinguish the onset of freezing and study the pathogenesis of FOG from the perspective of locomotor network dynamics. The described platform features high temporal and spatial resolution, and provides a set of effective data analysis methodologies to facilitate exploration of the dynamic electrophysiological patterns underlying FOG.

## Methods

### Patients and Surgical Procedure

Eight patients were enrolled in the study from January 2019 to June 2020. All patients underwent subthalamic nucleus-deep brain stimulation (STN-DBS) surgery in the Department of Neurosurgery of Beijing Tiantan Hospital (Table 1). All patients provided informed consent and signed the operation informed consent form. This study was approved by the Ethics Committee of Beijing Tiantan Hospital (No. KY 2018-008-02).

**TABLE 1:** Preoperative characteristics of the eight patients.

No. of patients	8
Sex	3M/5F
Age at time of surgery (Years)	
Range/Mean ± SD	52–73/62.63 ± 7.60
Age at disease onset (Years)	
Range/Mean ± SD	37–65/52.25 ± 9.15
Disease duration (Years)	
Range/Mean ± SD	7–15/10.38 ± 2.45
Dose of levodopa equivalent medication (mg/d)	
Range/Mean ± SD	488–1439.25/924.03 ± 356.49
Hoehn-Yahr Stage	
Range/Mean ± SD	2–3/2.56 ± 0.42
UPDRS III Score	
Range/Mean ± SD	32–79/52.88 ± 13.52
FOGQ Score	
Range/Mean ± SD	15–23/18.88 ± 2.75

As a routine clinical procedure, a Leksell stereotactic head frame was mounted onto the patient’s skull on the day of surgery under local anesthesia and was then aligned as closely as possible parallel to the anterior commissure–posterior commissure line. Then, an axial volumetric computed tomography (CT; slice thickness 0.625 mm, interslice gap 0 mm, 120 kVp) scan was taken. A General Electric 3.0 T magnetic resonance (MR) imaging scanner was used as a positioning scan (axial and coronal T1- and T2-weighted images with 1.0 mm slice thickness and no spacing). CT and MRI data were superimposed and fused in the surgical planning system (ELEKTA, Stockholm, Sweden). The surgeon confirmed the coordinates of the surgical target and the angle of trajectory based on MR images. Under local anesthesia, the surgical incision was made according to the calculated target coordinates, and a hole was drilled into the skull. After drilling, 8-contact cortical electrodes (Sinovation Medical Technology Co., Ltd., Beijing, China) were placed on the right sides parallel to the direction of the superior sagittal sinus. The electrode contacts covered the premotor area (PM), then the cortical electrode was fixed. The target position of the STN was confirmed by intraoperative microelectrode recording and electrode stimulation (L301; PINS Medical, Ltd., Beijing, China). Further details of this surgical procedure have been described previously (Meng et al., 2013). The position of the electrodes was examined again in a postoperative review, and any necessary adjustments were made after intracranial edema subsided. Through the open source software package, combined with preoperative MRI and postoperative CT images, the positions of the deep electrode contacts in the STN and the cortical electrode contacts could be determined (Hamilton et al., 2017; Horn et al., 2019).

### Recording Equipment

The EEG recording system (Nihon Kohden, Tokyo, Japan): the EEG recording system comprised a 64-channel electrode input box that can receive input signals from an external transducer. Cortical electrodes can be directly connected to the box, and through a customized connection cable, the STN electrode test cable of PD patients can also be connected to the box. The digital video system was used to record and view the patient’s video and the synchronized EEG waveform. NeuroWorkbench software was used to review, clip and export EEG data.

The Codamotion 3-D Movement Capture System (CODAMotion, Charnwood Dynamics Ltd., Rothley, Leicester): The codamotion system is an advanced three-dimensional motion capture system. Four cameras fixed on the bilateral roof capture the movements of nodes through active infrared wearable markers. If conditions permit, the system should be worn bilaterally according to the human bone model. The markers include the posterior superior iliac spine, thigh, shank, heel and fifth metatarsal. Codamotion provides convenient, efficient and accurate three-dimensional movement acquisition.

Deep brain stimulation electrode (L301; PINS Medical, Ltd., Beijing, China): the deep brain stimulation electrode comprises a 1.3-mm-diameter electrode with four stimulating contacts and four connecting contacts. The contact shape is cylindrical. The stimulating contact length is 1.5 mm and the stimulating contact spacing is 0.5 mm.

Subdural electrode (PSE-8A; Sinovation Medical Technology Co., Ltd., Beijing, China): the subdural electrode comprises eight stimulating contacts and an array of 1 × 8 electrode contacts. The silica gel sheet size is 8 × 80 mm, and contact spacing is 10 mm. The coverage of the subdural electrode includes the premotor, primary motor and sensory cortex.

### Synchronized Electrophysiology and Gait Data Acquisition

**FIGURE 1:**
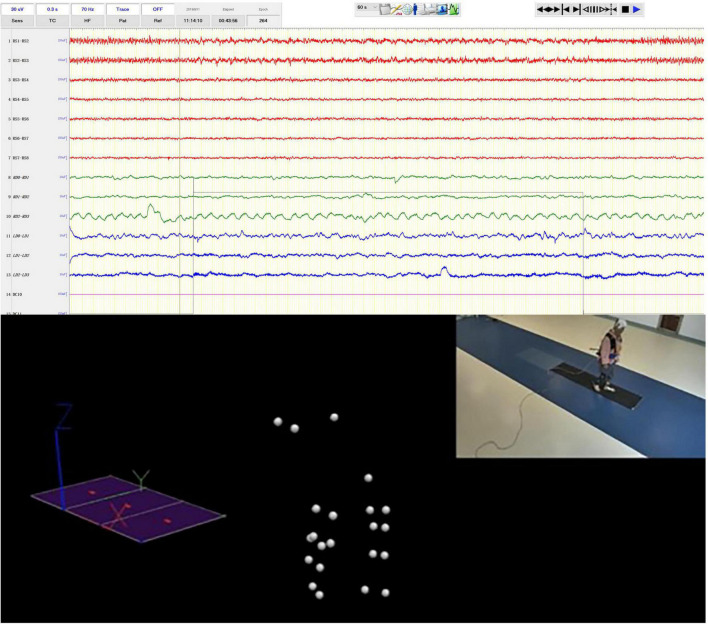
Syncronized iEEG recording and motion capture. The video clip in the upper right was simultaneously recorded by the video-EEG and real-time spatial positions of the optical sensitive nodes weared by the patients were captured by multiple surrounding cameras.

The electrophysiology acquisition parameters were a 0.08–660 Hz hardware filter and a sampling rate of 2000 Hz. A system reference averaging the electrical potentials of the fifth and sixth subdural electrodes was used during EEG acquisition. Electrical signal acquisition began 1–2 days after surgery. The trials were generally conducted during the OFF phase of the drug cycle (i.e., more than 12 h after the last anti-Parkinson’s drug was administered) (Bächlin et al., 2010). Electrical signals were collected when walking with or without cognitive load. The single record consisted of multiple repeated tasks, and the patient was prompted to start or stop the task by a random signal (Figure 1).

After the markers had been worn and the patient had adapted to a period of free activity, the gait data were collected. PD patients with FOG were asked to walk 5 m in an experimental area to collect gait features. The data were collected by the Codamotion 3-D Movement Analysis System with a sampling rate of 200 Hz.

To perform gait analysis, a number of steps need to be carried out in sequence. (1) Marker set: markers are required to complete a full lower body gait analysis. The different positions for clusters/marker drive boxes and markers are described in Figure 1. The markers can be classifying in two categories. Real markers are markers for which the 3D position is directly obtained from the position of a Codamotion active marker, and their spatial position data are used in current gait analysis. Virtual markers are markers for which their positions are obtained through computation. (2) Digitizing the pointer landmarks: a prompt window displays the name of the pointer that the experimenter needs to digitize. Once that pointer is digitized, the name of the following pointer is automatically displayed and the system waits for it to be digitized. (3) Visualizing the data: once calculation of the statistical data has been performed the user is able to see all of the results by selecting different layouts. (4) The raw data are exported in c3d format.

When the clinician presses the codamotion recording switch, a 5 V direct current signal is output, and the input signal is directly displayed on the interface of the EEG recording signal through the transducer. Similarly, when the clinician ends the gait signal recording, a 5 V TTL pulse is output and recorded by the EEG amplifier (Figure 1). Therefore, the collection of LFP and ECoG signals is synchronized with the collection of gait data.

### Gait Evaluation and Freezing of Gait Labeling

For the time up and go (TUG) test (Podsiadlo and Richardson, 1991; Herman et al., 2011), subjects wear flat shoes and sit on a chair (∼46 cm high) with their hands naturally resting on their legs. The ground is marked 5 m away from the seat and a sign is placed at this position. When the subject hears “start,” they are asked to stand up straight, walk at normal pace, turn around the sign, return to the seat and sit down, and then the timer is ended. No physical assistance can be given during the process. The time is recorded, and the subject can rest for 1 min between each test. Dual tasking (Fritz et al., 2015) that distracts patients’ attention allows FOG to be analyzed if the patient does not exhibit frozen gait during walking. Dual tasking usually consists of two tasks: primary motor or balance tasks (such as walking, standing) and secondary tasks required for distraction, such as simple computation tasks and language fluency tasks.

A PD expert manually labeled the freezing period by reviewing the synchronized high resolution video footage according to its definition, namely “brief, episodic absence or marked reduction of forward progression of the feet despite the intention to walk” (Heremans et al., 2013). The onset and ending of the FOG period were labeled.

### Machine Learning Approaches to Classified Freezing of Gait and Non-freezing of Gait

We used machine learning approaches to classified between FOG-epochs and non-FOG-epochs. From each of the 21 trials, we extracted 2 FOG epochs each lasted 2s and 4 same-length nFOG epochs, resulting in a total of 126 epochs (42 FOG epochs and 84 nFOG epochs). We extracted band power features (delta: 1–3 Hz, theta: 3–8 Hz, alpha: 8–13Hz, beta: 13–30 Hz, gamma: 30–60 Hz, and high-frequency oscillation: 60–300 Hz) from each epoch using a fast Fourier transform of 512 points. We employed a previously reported “mixed-effects random forest” (MERF) (Hajjem et al., 2014) to build classification models, which combines the linear mixed effects model and the random forest model, and is especially suitable for repeated measurement data. In building the model, we divided 70% of data into the training set and 30% of data into the testing set. Ten-fold cross validation was employed to cross-validate the classifier in the training set. Permutation test with 1,000 permutations was used to assess model robustness.

### Data Quality Manual Inspection

Collecting electrophysiological signals while the patient is walking is likely disturbed by motion artifacts. Therefore, before selecting each segment of electrical signal for analysis, we manually reviewed and scaled the signal quality, and selected level 1 or level 2 for subsequent analysis (Table 2).

**TABLE 2:** Classification of data quality.

Classification	Description
Level 1	The data quality is excellent in all time ranges, and there are almost no artifacts
Level 2	The data quality in most of the time range is excellent, with some artifacts, but the basic EEG waveforms are still reserved
Level 3	The data quality is acceptable in most of the time range, and the artifact interference is more obvious
Level 4	Most of the data is of poor quality and artifacts are obvious
Level 5	The entire signal is heavily contaminated by noise, and no normal EEG signal components can be seen

### Data Analysis and System Validation

**FIGURE 2:**
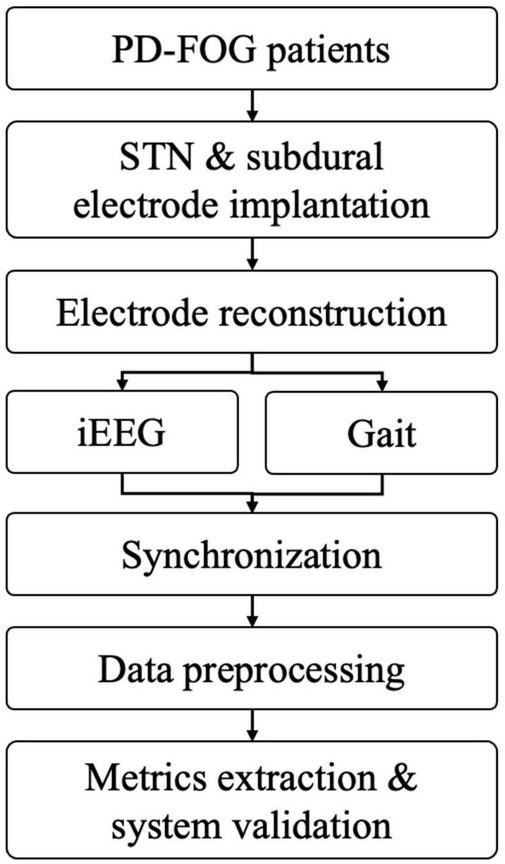
The working flow of current methodology (PD-FOG, Parkinson’s disease with freezing of gait; STN, subthalamic nucleus; iEEG, intracranial electroencephalogram).

After data inspection, interception and filtering, subsequent data analysis was performed, including FI calculation, band power analysis and coherence between cortical electrodes and STN (Figure 2).

Freeze index (FI) calculation: after quality control, we firstly calculated the FI with the hypothesis that FI would be significantly higher during the manually labeled FOG period. The FI was initially proposed by Moore et al. (2008) as the power ratio of the locomotor band (0.5–3.0 Hz) to the freeze band (3–8 Hz) derived from the frequency spectrum (Moore et al., 2008). A high FI is indicative of FOG. Originally the authors used vertical linear acceleration of the lower limb; however, in this study, we used spatial data on the vertical direction of the right heel. The reasons are as follows: the original version of the FI was based on acceleration data, however, in our system, the raw 3D trace was tracked by the high-speed camera. To generate the expected input of the FI algorithm, we calculated the second derivatives of the raw trace, representing instantaneous acceleration of the marker, from here on, we follow the original methods and parameters to calculate the FI. We used the erosion algorithm to correct the baseline, then, acceleration data was generated using the second derivative of the raw trace. Time frequency transformation was performed using the short-time fast Fourier method, then the FI was calculated. The FI values were compared between normal walking and FOG periods.

Band power analysis: firstly preprocessing of the electrophysiological data was performed, which included down-sampling to 1000 Hz (if the raw frequency rate was 2000 Hz), notch filtering of the 50 Hz line noise and its harmonics and re-referencing to bipolar montage before time frequency transformation. Any trials with an apparent artifact or drift were discarded. Time-frequency decomposition was carried out using a Morlet wavelet transform with frequencies of interest log-spaced between 1 and 170 Hz (38 total values). Subsequently, we performed averaging of power amplitude estimates within seven frequency bands: δ (1–3 Hz), θ (4–7 Hz), α (8–12 Hz), β1 (13–20 Hz), β2 (21–35 Hz), γ (36–69 Hz), and high γ (70–170 Hz). We then compared the power of the different frequency bands between two freezing and non-freezing conditions for each channel.

Coherence between cortical electrodes and STN: Pairwise coherence was calculated between bilateral STN depth electrodes and all cortical electrodes. The coherence was defined as below and the frequencies of interest were 1 to 200 Hz with a 1 Hz step length:


$$C\,o\,h\,e\,r\,e\,n\,c\,e=\frac{\left|E\,\left[S\,x\,y\right]\right|}{s\,q\,r\,t\,\left(E\,\left[S\,x\,x\right]*E\,\left[S\,y\,y\right]\right)}$$

where, Sxy and Sxx, Syy are estimates of the cross- and power-spectral densities (CSD/PSD). E[] denotes the average of the data segments. We firstly performed a statistical comparison between the raw electrophysiological data and shuffled surrogates to identify the significantly synchronized frequency band of interest. Then, based on the significant frequency band, coherence between FOG and non-FOG periods were further compared. To test the hemisphere difference, the coherence between ipsilateral STN/cortical pairs and contralateral STN/cortical pairs was also compared.

The codes used in this study can be found at: https://github.com/THIENC/DBS_FOG_Project_Analysis_Pipeline-master.

### Statistical Analysis

SPSS 23.0 software (IBM SPSS Statistics Inc., Chicago, IL, United States) was used for statistical analysis. Patient characteristics data are expressed as the mean ± standard deviation (x ± s). The paired *t*-test was used to calculate FI and Band Power between FOG and non-FOG periods (*P* < 0.05 was considered statistically significant).

## Results

### Patient Characteristics

All enrolled patients were clinically diagnosed as having primary PD and fit the following criteria: Hoehn–Yahr stage (Hoehn and Yahr, 1967) ≥ 2.0 in the drug-off period; the third item in the FOG questionnaire score (Giladi et al., 2000) ≥ 2.0; regarding the UPDRS-II score (Goetz et al., 2008), item 14 ≥ 2.0 and item 15 ≥ 2.0; displayed no major cognitive decline (Mini-Mental State Examination score (Folstein et al., 1983) ≥ 24 points). Recordings were made during walking for the eight patients (three males, five females; average age: 62.63 years, SD: 7.60 years). Clinical details are summarized in Table 1.

### Data Quality and Freezing of Gait Index

The eight patients completed a total of 41 walking tests, 30 of which had frozen episodes, and 21 of the 30 raw data were level 1 or 2 in quality (70%). The mean ± SD walking time for the TUG test was 85.94 ± 47.68 s (range: 38 to 190.14 s), the median was 67.32s; the mean ± SD freezing duration was 12.25 ± 7.35 s (range: 1.71 to 27.50 s), the median was 10.18s (Supplementary Table 1).

### Validation of CODA Data and the Freezing of Gait Index

**FIGURE 3:**
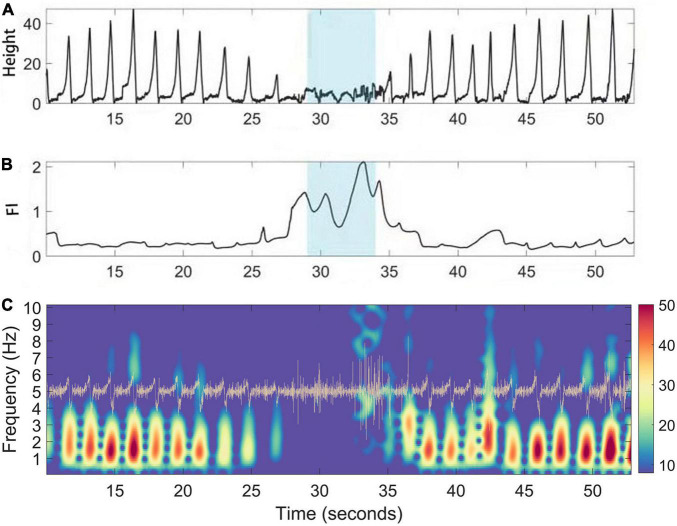
A case illustration of FI (Freeze index) increase during manually labeling freezing phase. **(A)** Gait positioning data (vertical *Z* axis) captured by the CODA system of PD patients during walking. **(B)** The corresponding dynamic fluctuation of FI; Blue area indicates the occurrence time of FOG determined manually. **(C)** Time frequency representation of the acceleration (trace in yellow) of the raw trace during walking of PD patients. During freezing, the power of the “locomotion band” (0–3 Hz) decreased.

During walking, the CODA system captured the position track of multiple active markers, and we chose the position track of the heel for demonstration and FI analysis. The illustrative case example (Figure 3A) showed the change in the *Z*-axis of the heel marker during the walking test. The height of steps decreased around 29–34 seconds, suggesting that FOG had occurred. As expected, the FI increased during the same period (Figure 3B). The acceleration data was generated by taking the derivatives of the raw trace (Figure 3C).

**FIGURE 4:**
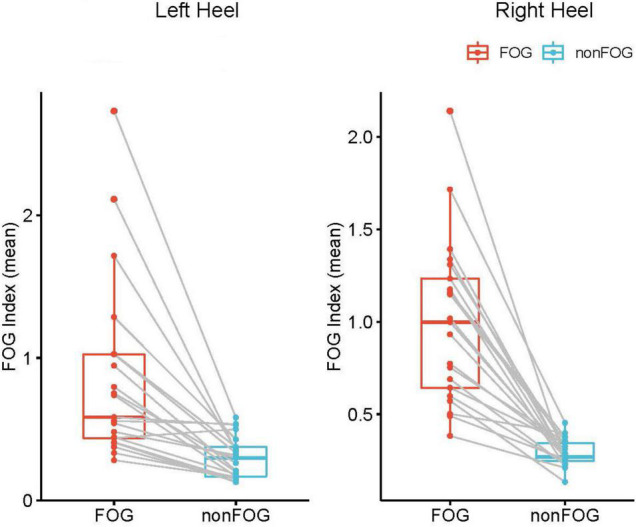
Statistical comparison of the FI between freezing the non-onset period at trial level. Each red data point represents the FI during the FOG of a walking trial, and blue data point represents the FI during the non-FOG of a walking trial. FI during FOG is significantly higher than effective walking period. Both sides exhibited similar results (*P*<0.05).

Between the different walking states (freezing or non-freezing), the paired *t*-test was conducted for the FI, and the statistical results showed that the FI values in the FOG period were statistically higher, as expected (*P* < 0.05), and this was replicable between each side of the data (Figure 4).

### Band Power Analysis of Electrocorticography/ Local Field Potential

**FIGURE 5:**
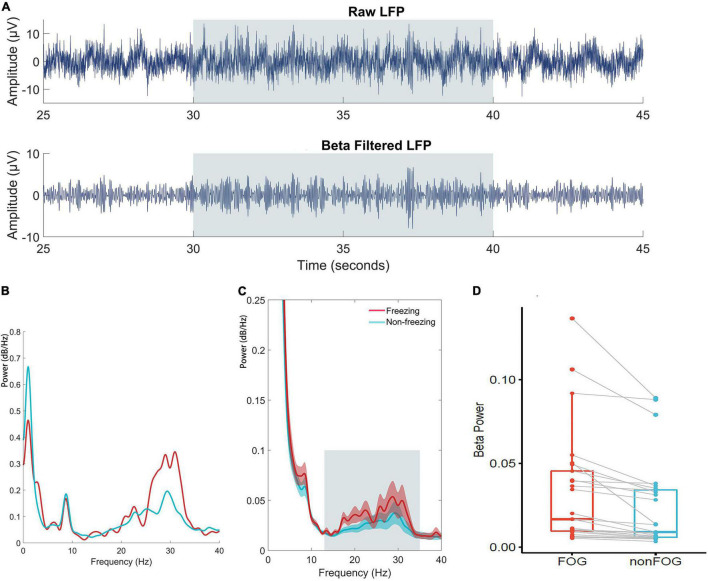
**(A)** Time series of raw and beta band filtered LFP. **(B)** An individual case illustration of power spectrum density of LFP in the STN between freezing and non-freezing period; The red line represents the power spectrum of LFP during FOG, and the blue line represents the power spectrum of LFP during non-FOG. **(C)** The group analyze of power spectrum density averaged by all included walking epochs. The shaded error bar indicates SEM and the gray square indicates beta range. **(D)** Statistical comparison of averaged beta band power of LFP between conditions at trial level indicated significantly increased beta power during freezing (*P*<0.05).

In the band power analysis, we compared the power of different frequency bands as described in the methods for each subdural and depth electrode between effective walking and FOG. The data from ECoG were all negative, indicating no significant band power difference between the freezing and non-freezing states. However, for the LFP from the depth electrodes, beta band power was significantly increased (*P* < 0.05) during freezing, and this was clear even at the individual level (Figure 5).

### Connectivity Between Cortical Regions and Subthalamic Nucleus

**FIGURE 6:**
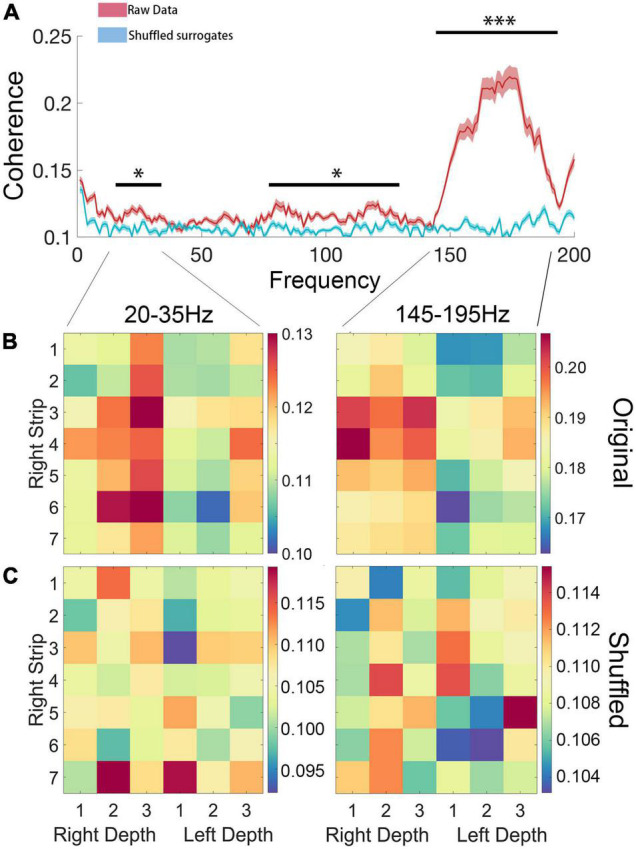
**(A)** Coherence of ipsilateral ECoG and LFP. Compared with shuffled surrogates, significantly higher coherence (*P* < 0.05, FDR correction) was found in high beta (20–35 Hz) and high gamma (145–195 Hz) bands. **(B,C)** Statistical comparison between original data and shuffled surrogates in high beta (20–35 Hz) and high gamma (145–195 Hz) bands, in which the rows and columns of matrix entries represent ECoG and LFP channels (“Right Strip 1–7” represent each channel of the ECoG, “Right Depth 1–3” represent each channel of the LFP of right STN and “Left Depth 1–3” represent each channel of the LFP of left STN) and the color scheme indicates the significant level of coherence (*P < 0.05; ***P < 0.001).

Pairwise coherence was calculated between bilateral STN depth electrodes and all cortical electrodes. Compared with shuffled surrogates, significantly higher coherence (cluster-based permutation test, *P* < 0.05, FDR correction) was found in high beta (20–35 Hz) and high gamma (145–195 Hz) bands (Figure 6). However, no coherence difference was found between walking states, which might be attributed to the short freezing period in each epoch.

**FIGURE 7:**
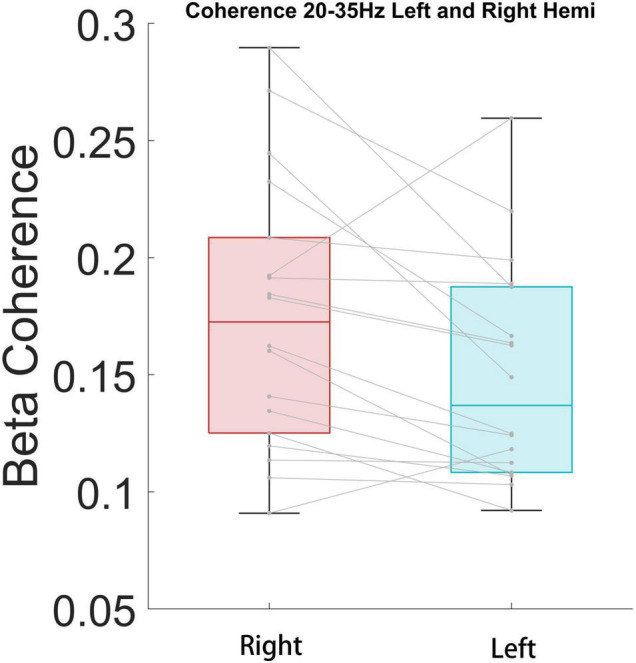
High beta coherence is significantly higher for ipsilateral STN/cortical pairs than contralateral pairs (*P* < 0.05).

We further tested whether there was a side preference of the connectivity between the STN and cortical areas for each significant band. To test the hemisphere difference, the coherence between bilateral STN and the right hemisphere ECoG of high beta and high gamma bands was compared. The high beta coherence of ipsilateral STN-cortical pairs was stronger than the contralateral equivalent (*P* < 0.05), but the high gamma coherence showed no difference (*P* = 0.42) (Figure 7).

### Machine Learning Classification

**FIGURE 8:**
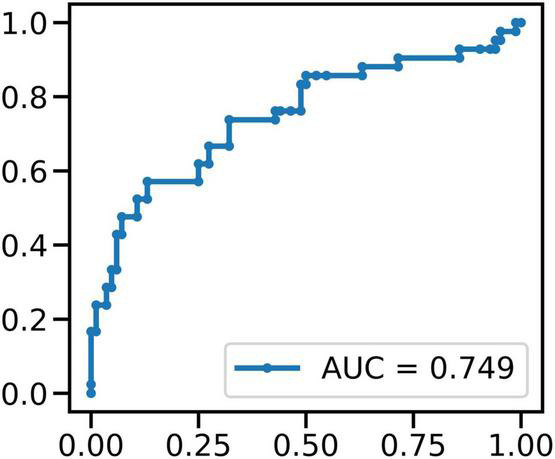
The result of MERF to classify between FOG-epochs and non-FOG-epochs: in a total of 126 epochs (containing 42 FOG epochs), STN LFP band power features showed above-chance performance (*p* < 0.01, permutation test) in identifying FOG epochs.

We used MERF to classify between FOG-epochs and nFOG-epochs. In a total of 126 epochs (containing 42 FOG epochs), STN LFP band power features showed above-chance performance (*p* < 0.01, permutation test) in identifying FOG epochs, rending an accuracy of 77% and an area under the receiver operating characteristic curve (AUC) of 0.75 (Figure 8).

## Discussion

Freezing of gait (FOG) is a common pathological gait in PD patients. It is widely believed that dysfunction of the basal ganglia circuit and related locomotor system is key in the occurrence of FOG in PD (Snijders et al., 2016). The study of the abnormal neural signature in patients with PD-FOG is valuable in revealing the mechanism of FOG in patients with PD, and thereby for seeking a new treatment model and guiding clinical DBS surgery.

Imaging studies have shown that there are structural and metabolic abnormalities in several brain regions in the basal ganglia–cortical circuit in PD-FOG patients. Pietracupa et al. (2018) compared PD-FOG patients and non-FOG PD patients (PD-nFOG) by MRI and found that the thickness of the gray matter of the frontal lobe, motor cortex and cingulate gyrus was decreased in PD-FOG patients. Canu et al. (2015) applied diffusion tensor imaging (DTI) combined with functional magnetic resonance imaging (fMRI) and observed that the functional connectivity of multiple brain areas, including the primary motor cortex, supplementary motor area and premotor area, is weakened in PD-FOG patients. Imaging studies have innate limitations because of the low time resolution; however, electrophysiological studies on the mechanisms in FOG patients, are still lacking at this stage. Storzer et al. (2017) pointed out that the occurrence of FOG has an electrophysiological basis in the basal ganglia region. The LFP in the STN region of PD-FOG patients shows a significant energy difference between the low β frequency band and the high β frequency band. Neurons in the STN area of PD-FOG patients showed different firing patterns in the two different states of riding and walking, suggesting functional differences depending on sensory feedback. Chen et al. (2019) recorded the LFP from the STN while PD patients performed single-task gait or walked while dual-tasking, which demonstrated that low beta and theta band oscillations within the STN area occur during gait susceptible to freezing in PD. However, there is still a lack of evidence at the circuit level. We implanted the STN deep electrode and the cortical electrode covering the primary motor and other areas in PD-FOG patients, and analyzed the LFP, ECoG, and gait data recorded simultaneously in the patient’s walking and freezing gait states.

In addition, Brain-Computer Interface (BCI) is a hot topic in neuroscience. Closed loop BCI is one of the main research directions at present. Through the feedback of biological signals, BCI can apply stimulus signals to the research object, so as to improve its external behavior, namely adaptive DBS. In this experimental platform, electrodes were directly implanted into the motor cortex and the STN, aiming to collect feedback signals through multiple targets. Subsequently, the feedback of biological signals could be further combined with DBS treatment, so that the startup and parameter setting of DBS could be regulated according to the onset of FOG. In other words, the patient’s movement performance is fed back to BCI as a biological signal, and then adaptive DBS is realized.

In this study, we first verified the reliability of the CODA system to assist in determining the period of FOG. The gait data collected by the CODA system in all channels can be directly decomposed into spatial three-dimensional coordinates. FOG is a type of gait disorder characterized by periodic and sudden gait delays and stops, lasting from a few seconds to a few minutes, which is manifested as a sudden decrease in the *Z*-axis on the time domain diagram (Figure 3A). This is also consistent with our manual labeling of the period of occurrence of FOG by reviewing the synchronized high resolution video footage. Previously, the FI was proposed to reflect the severity of FOG Moore et al. (2008). We also calculated the FI during the selected freezing period for system validating purposes. As expected, the FI during the freezing period was significantly higher than during the non-freezing period, indicating that the CODA system is reliable in assisting judgment of the freezing period (Figure 4).

After verifying the stability and reliability of the CODA system to determine the occurrence of FOG, we focused on the ECoG and LFP of the FOG period and the non-FOG period during walking. We selected each segment of electrical signal reaching level 1 or 2 for analysis, and of all the data collected in this study, more than 70% have limited motion artifacts, which also confirms the stability of our data collection platform.

We next performed band power analysis (Figure 5). The results suggested that the beta power of STN LFP during the FOG period was significantly higher than that of the non-FOG period, which was consistent with previous literature (Storzer et al., 2017; Chen et al., 2019). However, the ECoG spectrum did not exhibit a statistical difference between walking states, which may be attributed to insensitivity for fast dynamics of spectrum metrics. Previous literature confirmed structural and functional connectivity between the cortex and the basal ganglia in the resting state of PD patients (Tard et al., 2015; Mi et al., 2017; Bharti et al., 2019, 2020). Our study preliminarily confirmed that the coupling between the bilateral STN and the right cortical motor area, represented by the beta and high gamma band coherence, increased during walking (Figure 6), and high beta coherence is significantly higher for ipsilateral STN/cortical pairs than contralateral pairs (Figure 7). However, there was no significant difference between the FOG and non-FOG periods. The reason for this may be that FOG is a transient process, and the calculation of coherence required repeatedly measured trials to form stable results, thus lacking trial level dynamic sensitivity.

To improve the analysis the difference between the coherence during FOG/No-FOG periods, we used machine learning approaches to classified between FOG-epochs and non-FOG-epochs. After signal preprocessing, feature extraction and machine learning algorithm modeling, we used MERF algorithm for training and found all band power of LFP features showed above-chance performance (*p* < 0.01, permutation test) in identifying FOG epochs. This preliminary result suggests that the subsequent real-time monitoring of FOG episodes in patients’ daily life can provide quantitative and reliable reference for doctors’ diagnosis and treatment. In addition, the number of patients included was small, which may bias the results of machine learning classification even when MERF model was employed. But since this is a preliminary report stressing mainly the methodological platform of synchronized intracranial electrical activity and gait recording, further large cohort study adopting this approach reporting detailed clinical and electrophysiological outcomes will be conducted in the future.

This study has some limitations. First, the number of patients was small, and the sample size needs to be expanded in the future; Second, only the medicine-off state was evaluated; Third, the influence of motion on the electrophysiological signal is inevitable, but we tried to fix the collection line on the patient as much as possible to reduce external interference.

## Conclusion

This study aimed to describe a feasible synchronized recording system including intracranial EEG and gait data during walking. We verified the stability and reliability of the current system. In addition, preliminary results confirmed the stronger LFP beta power of STN during FOG and the functional connection between the cortex and STN in FOG patients. In the future, further exploration of the electrophysiological biomarker and neural substrate underlying FOG could be performed based on the current platform, which may provide a theoretical basis for optimizing and perfecting the development of DBS in the treatment of PD and the development of new neuromodulation technologies.

## Data Availability Statement

The original contributions presented in the study are included in the article/Supplementary Material, further inquiries can be directed to the corresponding author.

## Ethics Statement

The studies involving human participants were reviewed and approved by the Ethics Committee of Beijing Tiantan Hospital. The patients/participants provided their written informed consent to participate in this study.

## Author Contributions

D-FL and H-GL performed the surgery. D-FL and B-TZ analyzed the data and wrote the manuscript. D-FL, Y-YL, Y-TB, YJ, and XZ participated in the data collection. J-GZ, D-FL, B-TZ, H-GL, G-YZ, L-S, HZ, and A-CY designed the topic. J-GZ supervised the research process and modified the manuscript. All authors contributed to the article and approved the submitted version.

## Conflict of Interest

The authors declare that the research was conducted in the absence of any commercial or financial relationships that could be construed as a potential conflict of interest.

## Publisher’s Note

All claims expressed in this article are solely those of the authors and do not necessarily represent those of their affiliated organizations, or those of the publisher, the editors and the reviewers. Any product that may be evaluated in this article, or claim that may be made by its manufacturer, is not guaranteed or endorsed by the publisher.
