# Supplementary material for: Synchronized Intracranial Electrical Activity and Gait Recording in Parkinson’s Disease Patients With Freezing of Gait
Source: Front Neurosci. 2022 Mar 3;16:795417. doi: 10.3389/fnins.2022.795417 (PMC8927080; doi:10.3389/fnins.2022.795417)
Supplement: Supplementary file 1 [file Table_1.DOCX]

Table1 of supplementary materials: The characteristics of the FOG time.

| 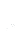ID | No.of  Test | total time of TUG test (s) | the time of FOG onset (s) | the time of FOG stopped (s) | freezing duration (s) |
| --- | --- | --- | --- | --- | --- |
| FOG01 | 1 | 67.3 | 30.2 | 40.8 | 10.6 |
|  | 2 | 57.4 | 29.2 | 35.0 | 5.8 |
|  | 3 | 89.1 | 20.4 | 29.4 | 9.0 |
| FOG002 | 4 | 38.1 | 10.4 | 19.4 | 9.0 |
|  | 5 | 47.0 | 11.4 | 18.5 | 7.1 |
|  | 6 | 48.0 | 14.2 | 24.4 | 10.2 |
| FOG03 | 7 | 111.7 | 40.0 | 67.5 | 27.5 |
|  | 8 | 58.6 | 19.4 | 30.3 | 10.9 |
| FOG04 | 9 | 49.0 | 13.2 | 30.6 | 17.4 |
|  | 10 | 38.0 | 11.5 | 24.3 | 12.8 |
|  | 11 | 38.0 | 9.1 | 27.1 | 18.0 |
| FOG05 | 12 | 164.2 | 59.8 | 61.5 | 1.7 |
|  | 13 | 190.1 | 32.8 | 39.6 | 6.8 |
| FOG06 | 14 | 52.0 | 14.1 | 23.0 | 8.9 |
|  | 15 | 38.0 | 9.8 | 178.0 | 8.2 |
| FOG07 | 16 | 79.1 | 5.0 | 10.0 | 5.0 |
| FOG08 | 17 | 98.0 | 6.9 | 17.9 | 11.0 |
|  | 18 | 116.9 | 8.1 | 30.4 | 22.3 |
|  | 19 | 140.5 | 7.6 | 33.0 | 25.4 |
|  | 20 | 120.5 | 4.0 | 9.3 | 5.3 |
|  | 21 | 163.0 | 28.4 | 52.7 | 24.3 |
|  | mean ± SD | 85.94 ± 47.68 |  |  | 12.25 ± 7.35 |
|  | median | 67.32 |  |  | 10.18 |
